# Different Growth Promoting Effects of Endophytic Bacteria on Invasive and Native Clonal Plants

**DOI:** 10.3389/fpls.2016.00706

**Published:** 2016-05-24

**Authors:** Zhi-Cong Dai, Wei Fu, Ling-Yun Wan, Hong-Hong Cai, Ning Wang, Shan-Shan Qi, Dao-Lin Du

**Affiliations:** ^1^Institute of Environment and Ecology & Academy of Environmental Health and Ecological Security, School of the Environment and Safety Engineering, Jiangsu UniversityZhenjiang, China; ^2^Key Laboratory of Modern Agricultural Equipment and Technology, Ministry of Education and Jiangsu Province, Jiangsu UniversityZhenjiang, China; ^3^Jingjiang College, Jiangsu UniversityZhenjiang, China

**Keywords:** repeatable aseptic culture system, bio-invasion, clonal plant, plant–microbe interaction, endophytic bacteria

## Abstract

The role of the interactions between endophytes and alien plants has been unclear yet in plant invasion. We used a completely germ-free culture system to quantify the plant growth-promoting (PGP) effects of endophytic bacteria *Bacillus* sp. on aseptic seedlings of *Wedelia trilobata* and of its native clonal congener *W. chinensis*. The endophytic bacteria did not affect the growth of *W. chinensis*, but they significantly promoted the growth of *W. trilobata*. With the PGP effects of endophytic bacteria, relative change ratios of the clonal traits and the ramets’ growth traits of *W. trilobata* were significantly greater than those of *W. chinensis*. Our results indicate that the growth-promoting effects of endophytes may differ between invasive and native clonal plants, and the endophytes of invasive plant may be host-specific to facilitate plant invasion.

## Introduction

In the past century, with the rapid development of global economic trade and cultural exchanges, a large number of plant species have broken their natural geographical barriers and have been introduced to new habitats (van Kleunen et al., 2015). Some of them have become successful invasive plant, which naturalize successfully and cause damage to ecosystem, economy and society (Bai et al., 2013). Therefore, understanding the mechanisms of plant invasion is dramatically important and contributes to their control management.

Plants may harbor abundant microorganisms in rhizosphere, rhizoplane, and endosphere (Hardoim et al., 2008; Compant et al., 2010). Soil biota is critical to enhance the plant’s capability of achieving resources from soil (Peiffer et al., 2013). Therefore, interactions between invasive plant and soil biota are hot topics in ecological researches. Rhizosphere microbiota is usually recommended as drivers in successful plant invasions owing to their plant growth promoting effects (Reinhart and Callaway, 2006; Coats and Rumpho, 2014). Invasive plants may change soil biota community to facilitate the plants’ invasion (Si et al., 2013), known as the “plant-soil feedback hypothesis” (Klironomos, 2002). This hypothesis suggests that facilitating effect could be achieved by encountering strong mutualism (Sun and He, 2010), by being released from soil-borne enemies (Callaway et al., 2004), or by inhibiting beneficial soil biota of native plants (Bozzolo and Lipson, 2013). Another important hypothesis involving the interactions of rhizosphere microbes and invasive plants, enhanced mutualisms hypothesis suggests that invasive plants may acquire better soil mutualists in their introduced ranges to enhance their competition ability or disrupting beneficial soil mutualists of native plants (Reinhart and Callaway, 2006). However, previous studies involving enhanced mutualisms hypothesis focused on the roles of rhizosphere microbiota on invasive plants (Sun and He, 2010).

Another important type of soil mutualist biota, endorhizosphere microbiota (abbreviated as endophytes), which live inside plants for at least part of their life cycle without causing any obvious symptoms (Hardoim et al., 2008), may also promote plant invasion like rhizosphere (Rout et al., 2013). It has been reported that endophytic bacteria may enhance the invasion ability of *Sorghum halepense* by changing soil biogeochemistry (Rout and Chrzanowski, 2009; Rout et al., 2013).

Many studies have investigated the interaction between microbes (e.g., rhizosphere microbes or endophytes) and invasive plants (Rout and Chrzanowski, 2009; Sun and He, 2010; Rout et al., 2013; Li et al., 2014). However, they did not use complete aseptic seedlings as studied material, which might cause a bias due to the interference of intrinsic endophytes in plants. Moreover, the behavior and ecological roles of rhizosphere microbes and endophytes for exotic plants’ invasion may vary across different environmental conditions (Long et al., 2008; Rout and Callaway, 2012) due to the potential inferences of different soil chemistry and soil biota (Reinhart and Rinella, 2016). Therefore, it is of great importance to use a uniform aseptic culture system to explore the interactions of plant–symbiont or plant-rhizosphere microbiota, in order to understand the mechanisms of plant invasion.

Here, we explore the interactions between endophytic bacteria and invasive plant by using a completely sterile pure culture system of repeatable conditions for invasive clonal plant *Wedelia trilobata* and its endophytic bacteria. We isolated the endophytic bacteria of *W*. *trilobata* and compared the promoting effects of the endophytic bacteria on aseptic seedlings of *W*. *trilobata* and its native congener *W*. *chinensis*. We aim to test whether the promoting effects of the endophytic bacteria are different on the aseptic seedlings of invasive *W*. *trilobata* and native *W*. *chinensis*.

## Materials and Methods

### Plant Materials

**FIGURE 1:**
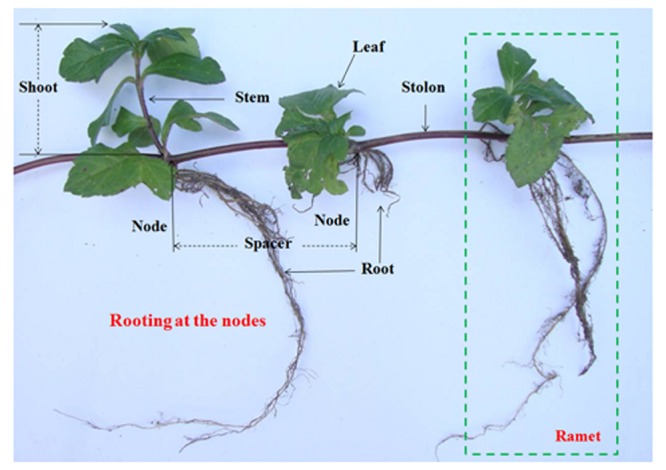
**Clonal fragment of *W. trilobata* plant**.

*Wedelia trilobata* (L.) Hitchc. (Asteraceae), native to tropical America, is one of the 100 worst invasive species in the world (IUCN, 2001). *W. trilobata* spreads rapidly by strong stolon growth (**Figure 1**) and often overgrows with thick litter layer (Qi et al., 2014a). It is notorious to natural ecosystems in South China (Qi et al., 2014b). *W. trilobata* plants were randomly collected from its invading habitat Haikou, China. *W. chinensis* (Osbeck.) Merr. (Asteraceae) is the native congener of *W. trilobata* in China (Song et al., 2010). Both *W. trilobata* and *W*. *chinensis* are typical clonal plant. These two *Wedelia* plants were propagated in a greenhouse at Jiangsu University, Zhenjiang, China (Dai et al., 2016). The work has been conducted in conformity with the ethical standards of the field, and did not involved human subjects or animals.

### Endophytic Bacteria Strain Isolation from *W. trilobata*

The stems near root (∼3 cm) of healthy *W. trilobata* were collected and were cleaned with running tap water. Under sterile conditions, the stems samples were surface sterilized by stepwise washing in 70% ethanol for 1 min, rinsing with sterile water two times, and then with sodium hypochlorite solution (2% available Cl) for 10 min, followed by five rinses in sterile distilled water. A 100 μl sample of distilled water from the final rinse was planted on Luria–Bertani (LB) agar (Sambrook and Russell, 2001) to confirm that the disinfection process was successful.

After surface disinfection, the stem tissues were cut into approximately 0.5 cm pieces, then slit into two pieces. The wound was stuck to solid LB medium plate. The plates were incubated at 30°C and monitored daily for bacterial colony development over 5 days. Bacterial colonies were isolated and purified by streaking and selection based on phenotypic characteristics, e.g., colony color and morphology (Gagne-Bourgue et al., 2013).

### Cell Morphology Observation of Endophytic Bacteria of *W. trilobata* by SEM

The endophytic bacteria (50 ml LB liquid medium culture in 250 ml triangular flasks) were incubated separately at 30°C with shaking (200 rpm) for 16 h. After centrifugation at 10,000 rpm for 15 min, the substrate cells were harvested and washed three times with phosphate buffer solution (PBS, pH 7.2). The collected cells were fixed with 2.5% glutaraldehyde at 4°C for 24 h, then washed three times with PBS. After eliminating solution, the dehydration process was conducted with 30, 50, 70, 80, and 95% of alcohol for 15 min each step, and 100% of alcohol for two times (Ahmad Barudin et al., 2014). After freeze drying for 12 h in the vacuum freeze dryer (Lyoquest-55, Azbil Telstar Technologies S.L.U. Spain), the bacterial cells were harvested and coated with gold under vacuum for examination by a scanning electron microscope (SEM) (S-3400N, HITACHI, Japan) with an acceleration voltage of 10 kV.

### Phylogenetic Analysis of Endophytic Bacteria of *W. trilobata*

Bacterial genomic DNA was extracted from pure cultures using the Microbial DNA Isolation Kit (MoBio). The universal bacterial primers, Bac8F (5′-AGA GTT TGA TCC TGG CTC AG-3′) and 1492R (5′-GGT TAC CTT GTT ACG ACT T-3′) (Fierer and Jackson, 2006), were used to amplify the 16Sr-DNA. The DNA PCR amplification was performed with initial denaturing at 94°C (5 min), followed by 30 PCR cycles of 1 min denaturing at 95°C, 1 min annealing at 54°C, 1 min extension at 72°C and ended with 10 min extension at 72°C. The PCR products were subjected to electrophoresis on 1.5% agarose gels in 1× TBE buffer and stained with ethidium bromide to verify the target size. The PCR products were purified to remove non-target products using the PCR purification kit (Axygen Bioscience Inc., USA), and then sequenced by Sangon Biotech (Shanghai) Co. Ltd., (China). The sequences were subjected to a BLAST search^1^, aligned, and built phylogenetic tree using MEGA 6 with neighbor–joining (NJ) method (Dai et al., 2015).

### Aseptic Culture System

In this study, an aseptic culture system, which contained uniform aseptic seedlings and sterile culture environment of nutrient control, was used to investigate the effects of endophytic bacteria on invasive plant. Aseptic seedlings were produced by fresh sprouts in ramets of *W. trilobata* and *W. chinensis*. Fresh apical buds of *W. trilobata* and *W. chinensis* were surface-sterilized with 5% sodium hypochloride solution for 10 min and washed thoroughly five times with sterilized distilled water. Murashige and Skoog (MS) medium (Murashige and Skoog, 1962) supplemented with 0.8 mg⋅l^-1^ 6-benzylaminopurine, 0.1 mg⋅l^-1^ 1-naphthaleneacetic acid, and 0.8 mg⋅l^-1^ silver nitrate. Media were adjusted to pH 6.5 before sterilization by autoclaving for 20 min at 115°C. All the cultures were kept in a culture room at 24°C under a 16 h day and 8 h night photoperiod with 450 μmol⋅m^-2^⋅s^-1^. The aseptic seedlings were confirmed as complete aseptic seedlings using the coating plate method and 16S-rDNA PCR method (Data sheet 1.doc). These aseptic seedlings were subsequently used as explants, and cultured *in vitro* as follows.

Multiple axillary buds proliferated after approximately 50 days (**Supplementary Figure S1A**). Apical shoots (∼3 cm length) cut from axillary buds (**Supplementary Figure S1B**) were cultured in glass culture bottles (250 ml) containing 35 ml of MS medium for approximately 3∼5 days to obtain aseptic seedling with roots (**Supplementary Figure S1C**). Aseptic seedlings in similar sizes were transferred from MS medium into aseptic culture environment (**Supplementary Figure S1D**), which contained 150 g sterilized water-clean sand and 40 ml sterilized half-strength Hoagland liquid nutrient solution (Hoagland and Arnon, 1950) in an incubator (temperature: 28 ± 2°C; photoperiod: 16 h light and 8 h dark; light intensity: 450 μmol ⋅ m^-2^⋅s^-1^).

### Endophyte Experiments

The endophytic bacteria strain were grown in LB medium for 16 h (OD_660_ = 1) at 30°C with shaking (200 rpm). Then the endophytic bacteria cells were collected by centrifugation (13,000 rpm for 15 min at 4°C). Cells were re-suspended with sterile 0.05 mM PBS (pH 7.0) at a final concentration of 10^7^ CFU⋅ml^-1^ to uniform population of bacteria for seedlings inoculation (Taghavi et al., 2009).

After all aseptic seedlings grown stably for 2 days in the aseptic culture system, 2 ml phosphate buffer of the endophytic bacteria cells (E^+^) were added around the roots of aseptic seedlings of *W. trilobata* (*Wt*) and *W. chinensis* (*Wc*). Two milliliters of phosphate buffer of heat-killed endophytic bacteria cells were added as negative control treatment (E^-^). Thus, there were four treatments for two plant species and two endophytic bacteria addition treatments: (1) *Wc*E^-^, (2) *Wc*E^+^, (3) *Wt*E^-^, and (4) *Wt*E^+^. The colonization of endophytic bacteria in aseptic seedlings were identified using coating plate method and 16S-rDNA sequences method (Data sheet 2.doc). Each treatment was repeated five times. Six weeks after bacterial inoculation, the phenotypic growth (root #, root length, shoot diameter, shoot length), clonal growth (spacer #, spacer length), and dry biomass (the second pair of leaves mass, below and aboveground mass, total mass) were measured.

### Data Analysis

Root vs. shoot length/mass ratio were calculated to evaluate resource allocation strategy of *W. trilobata* and *W. chinensis*. Two-way ANOVAs were used to compare means of growth traits between treatments using Duncan’s multiple-range test (α = 0.05) in the endophyte experiments. To eliminate the potential interference of background value from the plant species, the relative change ratio (RCR) of the indices was also calculated as follow: RCR (%) = [(E^+^– E^-^)/E^-^] × 100%. One-way ANOVAs was performed to quantify the effects of endophytic bacteria on plants between *W. trilobata* and *W. chinensis*, using Duncan’s multiple–range test (α = 0.05).

## Results

### Identification of Endophytic Bacteria of *W. trilobata*

**FIGURE 2:**
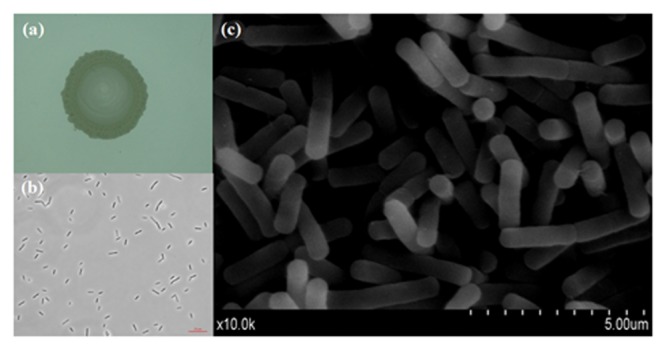
**Morphology of endophytic bacteria WtEB-JS040 of *W. trilobata*. (a)** Bacterial colony, **(b)** normal light microscope, **(c)** scanning electron microscope (SEM).

**FIGURE 3:**
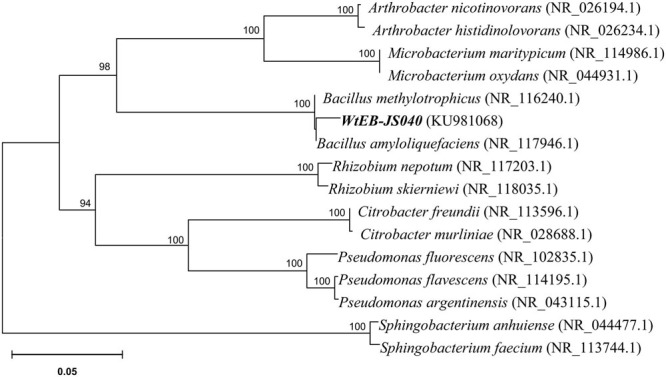
**Phylogenetic tree of endophytic bacteria WtEB-JS040 of *W. trilobata* based on 16Sr-DNA gene sequence**.

The endophytic strain isolated from *W. trilobata* and used in this study was identified as *Bacillus* sp. WtEB-JS040 (naming scheme: Wt – *Wedelia trilobata*, EB – endophytic bacteria, JS – Jiangsu; hereinafter abbreviated as JS040) based on its phenotypic characteristics (**Figure 2**) and 16Sr-DNA gene sequence (**Figure 3**; Supplementary Table S1; GenBank accession no. KU981068). A fragment (1,152 Bp) of 16Sr-DNA was amplified by PCR from strain JS040 (**Supplementary Figure S2**). Sequence analysis and homology comparison of 16Sr-DNA gene sequence showed that strain JS040 had a similarity of 99% with *Bacillus amyloliguefaciens* (GenBank no. NR_117946.1) and *Bacillus methylotrophicus* (GenBank no. NR_116240.1).

### Effects of JS040 on the Aseptic Seedling of *W. trilobata* and *W. chinensis*

**FIGURE 4:**
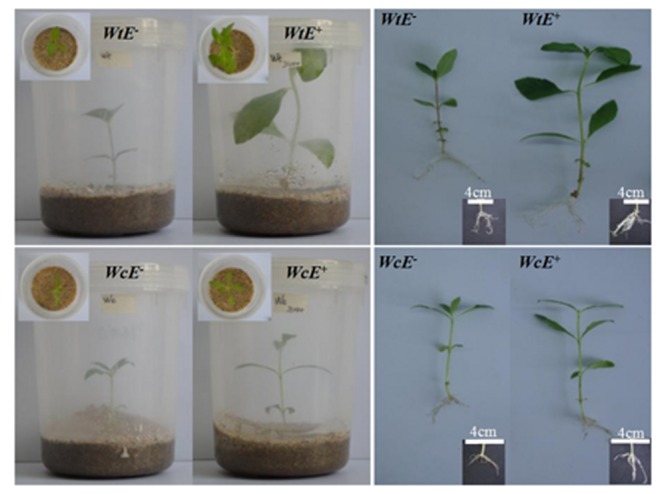
**The growth of *W. trilobata* (*Wt*) and *W. chinensis* (*Wc*) aseptic seedlings inoculated with (*E*^+^) / without (*E*^-^) WtEB-JS040 strain**.

**FIGURE 5:**
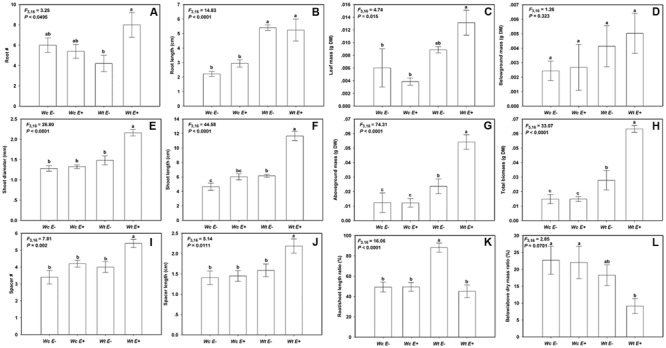
**The growth of *W. trilobata* (*Wt*) and *W. chinensis* (*Wc*) aseptic seedlings inoculated with (*E*^+^) / without (*E*^-^) WtEB-JS040 strain.** Growth phenotype: **(A)**-root number, **(B)**-root length, **(C)**-shoot diameter, **(D)**-shoot length; Clonal traits: **(E)**-spacer number, **(F)**-spacer length; Biomass: **(G)**-leaf mass, **(H)**-belowground mass, **(I)**-aboveground mass, **(J)**- total mass; Allocation strategy: **(K)**-root/shoot length ratio, **(L)**- root/shoot mass ratio. Different letters indicate significant growth difference of seedlings between the endophytes treatments. Bars represent standard errors (*n* = 5).

The endophytic bacteria JS040 inoculation showed significant promoting effects on *W. trilobata* (**Figure 4**). The phenotypic growth (root #, shoot diameter, and shoot length) (**Figures 5A,C,D**), clonal growth (spacer # and spacer length) (**Figures 5E,F**), and biomass (aboveground mass and total mass) (**Figures 5I,J**) of *W. trilobata* were greatly increased with the JS040 inoculation. However, JS040 reduced the resource cost in *W. trilobata* roots (**Figures 5K,L**). JS040 did not significantly change the growth phenotype, clonal growth, biomass and resource allocation in *W. chinensis* (**Figures 5A–L**).

**FIGURE 6:**
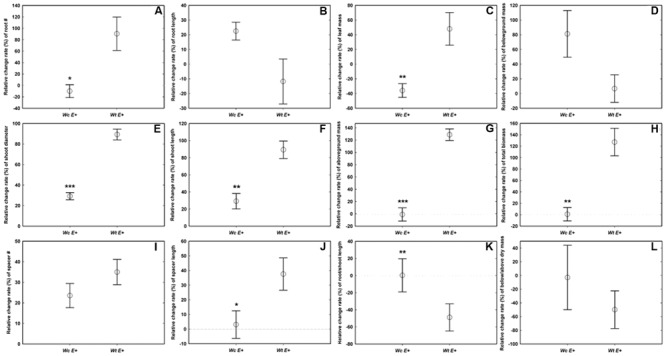
**The relative change ratio (RCR) of *W. trilobata* (*Wt*) and *W. chinensis* (*Wc*) aseptic seedlings inoculated with (*E*^+^) WtEB-JS040 strain.** Growth phenotype: **(A)**-root number, **(B)**-root length, **(C)**-shoot diameter, **(D)**-shoot length; Clonal traits: **(E)**-spacer number, **(F)**-spacer length; Biomass: **(G)**-leaf mass, **(H)**-belowground mass, **(I)**-aboveground mass, **(J)**- total mass; Allocation strategy: **(K)**-root/shoot length ratio, **(L)**- root/shoot mass ratio. ^∗^ (*p* < 0.05), ^∗∗^ (*p* < 0.01), and ^∗∗∗^ (*p* < 0.001) indicate the difference of the RCR of seedlings between the endophytes treatments. Bars represent standard errors (*n* = 5).

Compared with *W. chinensis*, JS040 significantly increased the RCR of root # (**Figure 6A**), shoot diameter (**Figure 6C**), shoot length (**Figure 6D**), spacer length (**Figure 6F**), leaf mass (**Figure 6G**), aboveground mass (**Figure 6I**), total mass (**Figure 6J**) of *W. trilobata*, whereas JS040 decreased the RCR of root vs. shoot of *W. trilobata* (**Figure 6K**) (**Table 1**).

**Table 1:** One-Way ANOVAs for the effects of endophytic bacteria WtEB-JS040 on the relative change rate of the growth of invasive *W. trilobata* and native *W. chinensis.*

Traits	Source	df_1_	df_2_	*F*	*P*
Growth phenotype	Root #	1	8	10.32	0.012*
	Root length	1	8	4.33	0.071
	Shoot diameter	1	8	44.47	0.0002***
	Shoot length	1	8	19.52	0.002**
Clonal traits	Spacer #	1	8	1.82	0.214
	Spacer length	1	8	5.64	0.045*
Biomass	Leaf mass	1	8	12.07	0.008**
	Belowground mass	1	8	0.07	0.802
	Aboveground mass	1	8	82.08	<0.0001***
	Total mass	1	8	22.22	0.002**
Allocation strategy	Root/shoot length ratio	1	8	19.22	0.002**
	Root/shoot mass ratio	1	8	3.71	0.090

*Significance levels: ^∗∗∗^*P* < 0.001, ^∗∗^*P* < 0.01, ^∗^*P* < 0.05.*

## Discussion

### Different Effects of Endophytic Bacteria WtEB-JS040 between *W. trilobata* and *W. chinensis*

The effects of soil biota on plants may be estimated inappropriately owing to the variation of culture environment gradients, plant genetics, and even soil biota across very small spatial scales (Reinhart and Rinella, 2016). In this study, we suggest using a completely aseptic culture system containing uniform aseptic seedlings and repeatable environment (**Supplementary Figure S1**), to explore the effects of microorganism on invasive plants. As this aseptic culture system eliminates other microorganism in growth media and intrinsic endophytes in target plants, our results contribute to our understanding of the real functions of microbes on plant invasion.

Previous studies have shown that auxin regulates initiation and emergence of root (Ljung et al., 2005; Overvoorde et al., 2010), and indoleacetic acid (IAA) synthesized by plant-associated bacteria plays a major role in the development of the host plant root system (Patten and Glick, 2002; Tchinda et al., 2016). In the present study, the endophytic bacteria JS040 showed significant promoting effects on its host invasive clonal plant *W. trilobata*. Although the root length and mass of *W. trilobata* did not increase after endophytes inoculation, endophytic JS040 increased the growth performance of *W. trilobata*, which might be due to the increase of the root number and vitality stimulated by bacteria-excreted auxin to enhance the nutrient uptake capability. Considering the fast dispersal of *W. trilobata* through clonal reproduction (**Figure 1**; Qi et al., 2014a; Si et al., 2014; Dai et al., 2016), the increase of shoot length, spacer number, spacer length (**Figures 5D–F**) will potentially enhance the expansion ability of ramet population of *W. trilobata*. In addition, we also found endophytic JS040 may help invasive *W. trilobata* to allocate less resource to below-ground system (**Figure 5K**) in the meantime of keeping its shoot growth dominance, which may be because that JS040 was isolated from stems.

As for the native congener *W. chinensis*, endophytic bacteria JS040 did not have significant promoting effects (**Figure 5**). After inoculation of JS040, the RCR of clonal traits (spacer length) and potential ramets’ growth traits (root #, shoot diameter and length, leaf mass, aboveground mass, total mass) of *W. trilobata* were higher than that of *W. chinensis* (**Figure 6**; **Table 1**), suggesting that the growth-promoting effects of endophytic bacteria JS040 may differ between invasive and native clonal plants. Therefore, our results indicate that endophytes of *W. trilobata* may be host-specific to increase the growth of *W. trilobata*, which provides the preliminary supports for enhanced mutualisms hypothesis (Reinhart and Callaway, 2006) from endosymbiosis using complete aseptic culture system (**Supplementary Figure S1**).

### Implications for Future Researches

The plant–microbe interactions are important in plant fitness and adaptability, which have evolved in direct association with microbes functioning as both agonists and antagonists in terms of plant development and defense activities (Coats and Rumpho, 2014). The interactions between plant and rhizosphere microbe have been well studied in plant invasion ecology (Klironomos, 2002; Callaway et al., 2004; Sun and He, 2010; Rout and Callaway, 2012; Si et al., 2013). However, our understanding of the complex interactions between endophytes and their host plants is not clear yet, and our finding of the promoting growth effect mediated by endophytes contributes to this area. This promoting growth effects could be important to the invasion process of alien invasive plants. Future works can be launched to investigate the interactions between invasive plant and endophytes as follows:

(1) The roles of more endophytes on host plants, endophytes from different tissues of host invasive plants and host native plants. These aspects make the cross inoculation experiments of endophytes from different host plants essential to understand the contributions of endophytes to plant invasion; (2) Microbial colonization of rhizoplane and endosphere in invasive plants. Efficient colonization in the roots of hosts is endophytes’ first step to function in the plant–microbe interactions (Long et al., 2008; Compant et al., 2010); (3) Mechanisms of endophytes promoting the growth of hosts and the hosts’ regulation of endophytes. In this way, we need to explore the feedback system between invasive plants and endophytes. Since researchers do not fully understand the mechanisms by which bacterial endophytes promotes the growth of host plant, we still need to conduct in-depth researches in the field of plant invasion. What is more, we shall pay more attention in exploring the regulation mechanisms of invasive plants on endophytes in future.

## Author Contributions

Z-CD, S-SQ, and D-LD designed the study. Z-CD, WF, and L-YW carried out the experiment work for strain isolation and identification. S-SQ and H-HC carried out the experiment work for aseptic seedlings culture. WF and NW carried out the experiment work for endophyte experiments and data selection. Z-CD and D-LD did the data analysis. Z-CD and S-SQ wrote the manuscript. All authors have read and approved the final version of the manuscript.

## Conflict of Interest Statement

The authors declare that the research was conducted in the absence of any commercial or financial relationships that could be construed as a potential conflict of interest.

## Footnotes

1 http://blast.ncbi.nlm.nih.gov/Blast.cgi

## Supplementary Material

FIGURE S1 **Aseptic culture system for endophytes research. (A)** multiple axillary buds, **(B)** apical shoots cut from axillary buds, **(C)** aseptic seedling with roots, **(D)** aseptic seedling system.

FIGURE S2 **Amplification of 16S-rDNA from WtEB-JS040 strain.** Lane M, DL2000 LADDER; Lane 1, 16S-rDNA fragment amplified by PCR.

## References

[B1] Ahmad Barudin N. H. Sreekantan S. Ong M. T. Lai C. W. (2014). Synthesis, characterization and comparative study of nano-Ag–TiO2 against Gram-positive and Gram-negative bacteria under fluorescent light. *Food Control* 46 480–487. 10.1016/j.foodcont.2014.05.046

[B2] Bai F. Chisholm R. Sang W. Dong M. (2013). Spatial risk assessment of alien invasive plants in China. *Environ. Sci. Technol.* 47 7624–7632. 10.1021/es400382c 23738912

[B3] Bozzolo F. H. Lipson D. A. (2013). Differential responses of native and exotic coastal sage scrub plant species to N additions and the soil microbial community. *Plant Soil* 371 37–51. 10.1007/s11104-013-1668-2

[B4] Callaway R. M. Thelen G. C. Rodriguez A. Holben W. E. (2004). Soil biota and exotic plant invasion. *Nature* 427 731–733. 10.1038/nature02322 14973484

[B5] Coats V. C. Rumpho M. E. (2014). The rhizosphere microbiota of plant invaders: an overview of recent advances in the microbiomics of invasive plants. *Front. Microbiol.* 5:368 10.3389/fmicb.2014.00368 PMC4107844 25101069

[B6] Compant S. Clément C. Sessitsch A. (2010). Plant growth-promoting bacteria in the rhizo- and endosphere of plants: their role, colonization, mechanisms involved and prospects for utilization. *Soil Biol. Biochem.* 42 669–678. 10.1016/j.soilbio.2009.11.024

[B7] Dai Z. C. Fu W. Qi S. S. Zhai D. L. Chen S. C. Wan L. Y. (2016). Different responses of an invasive clonal plant *Wedelia trilobata* and its native congener to gibberellin: implications for biological invasion. *J. Chem. Ecol.* 42 85–94. 10.1007/s10886-016-0670-6 26879680

[B8] Dai Z. C. Qi S. S. Miao S. L. Liu Y. T. Tian Y. F. Zhai D. L. (2015). Isolation of NBS-LRR RGAs from invasive *Wedelia trilobata* and the calculation of evolutionary rates to understand bioinvasion from a molecular evolution perspective. *Biochem. Syst. Ecol.* 61 19–27. 10.1016/j.bse.2015.05.004

[B9] Fierer N. Jackson R. B. (2006). The diversity and biogeography of soil bacterial communities. *Proc. Natl. Acad. Sci. U.S.A.* 103 626–631. 10.1073/pnas.0507535103 16407148 PMC1334650

[B10] Gagne-Bourgue F. Aliferis K. A. Seguin P. Rani M. Samson R. Jabaji S. (2013). Isolation and characterization of indigenous endophytic bacteria associated with leaves of switchgrass (*Panicum virgatum* L.) cultivars. *J. Appl. Microbiol.* 114 836–853. 10.1111/jam.12088 23190162

[B11] Hardoim P. R. van Overbeek L. S. Elsas J. D. V. (2008). Properties of bacterial endophytes and their proposed role in plant growth. *Trends Microbiol.* 16 463–471. 10.1016/j.tim.2008.07.008 18789693

[B12] Hoagland D. R. Arnon D. I. (1950). The water-culture method for growing plants without soil. *Univ. Calif. Agric. Exp. Stn.* 347 1–32.

[B13] IUCN (2001). *100 of the World’s Worst Invasive Alien Species*. Auckland: Invasive Species Specialist Group.

[B14] Klironomos J. N. (2002). Feedback with soil biota contributes to plant rarity and invasiveness in communities. *Nature* 417 67–70. 10.1038/417067a 11986666

[B15] Li H. Zhang X. M. Zheng R. S. Li X. Elmer W. H. Wolfe L. M. (2014). Indirect effects of non-native Spartina alterniflora and its fungal pathogen (Fusarium palustre) on native saltmarsh plants in China. *J. Ecol.* 102 1112–1119. 10.1111/1365-2745.12285

[B16] Ljung K. Hull A. K. Celenza J. Yamada M. Estelle M. Normanly J. (2005). Sites and regulation of auxin biosynthesis in *Arabidopsis* roots. *Plant Cell* 17 1090–1104. 10.1105/tpc.104.029272 15772288 PMC1087988

[B17] Long H. H. Schmidt D. D. Baldwin I. T. (2008). Native bacterial endophytes promote host growth in a species-specific manner; phytohormone manipulations do not result in common growth responses. *PLoS ONE* 3:e2702 10.1371/journal.pone.0002702 PMC2444036 18628963

[B18] Murashige T. Skoog F. (1962). A revised medium for rapid growth and bioassays with tabacco tissue cultures. *Physiol. Plant.* 15 437–439. 10.1111/j.1399-3054.1962.tb08052.x

[B19] Overvoorde P. Fukaki H. Beeckman T. (2010). Auxin control of root development. cold spring harb. *Perspect Biol.* 2 a001537–a001537. 10.1101/cshperspect.a001537 PMC2869515 20516130

[B20] Patten C. L. Glick B. R. (2002). The role of bacterial indoleacetic acid in the development of the host plant root system. *Appl. Environ. Microbiol.* 68 3795–3801. 10.1128/AEM.68.8.3795-3801.2002 12147474 PMC124051

[B21] Peiffer J. A. Spor A. Koren O. Jin Z. Tringe S. G. Dangl J. L. (2013). Diversity and heritability of the maize rhizosphere microbiome under field conditions. *Proc. Natl. Acad. Sci. U.S.A.* 110 6548–6553. 10.1073/pnas.1302837110 23576752 PMC3631645

[B22] Qi S. S. Dai Z. C. Miao S. L. Zhai D. L. Si C. C. Huang P. (2014a). Light limitation and litter of an invasive clonal plant, *Wedelia trilobata*, inhibit its seedling recruitment. *Ann. Bot.* 114 425–433. 10.1093/aob/mcu075 24825293 PMC4111383

[B23] Qi S. S. Dai Z. C. Zhai D. L. Chen S. C. Si C. C. Huang P. (2014b). Curvilinear effects of invasive plants on plant diversity: plant community invaded by *Sphagneticola trilobata*. *PLoS ONE* 9:e113964 10.1371/journal.pone.0113964 PMC4245253 25426856

[B24] Reinhart K. O. Callaway R. M. (2006). Soil biota and invasive plants. *New Phytol.* 170 445–457. 10.1111/j.1469-8137.2006.01715.x 16626467

[B25] Reinhart K. O. Rinella M. J. (2016). A common soil handling technique can generate incorrect estimates of soil biota effects on plants. *New Phytol.* 210 786–789. 10.1111/nph.13822 26738893

[B26] Rout M. E. Callaway R. M. (2012). Interactions between exotic invasive plants and soil microbes in the rhizosphere suggest that ‘everything is not everywhere.’ *Ann. Bot.* 110 213–222. 10.1093/aob/mcs061 22451600 PMC3394644

[B27] Rout M. E. Chrzanowski T. H. (2009). The invasive *Sorghum halepense* harbors endophytic N2-fixing bacteria and alters soil biogeochemistry. *Plant Soil* 315 163–172. 10.1007/s11104-008-9740-z

[B28] Rout M. E. Chrzanowski T. H. Westlie T. K. DeLuca T. H. Callaway R. M. Holben W. E. (2013). Bacterial endophytes enhance competition by invasive plants. *Am. J. Bot.* 100 1726–1737. 10.3732/ajb.1200577 23935109

[B29] Sambrook J. Russell D. W. (2001). *Molecular Cloning: A Laboratory Manual*. New York, NY: Cold Spring Harbor Laboratory Press (CSHL) press.

[B30] Si C. C. Dai Z. C. Lin Y. Qi S. S. Huang P. Miao S. L. (2014). Local adaptation and phenotypic plasticity both occurred in *Wedelia trilobata* invasion across a tropical island. *Biol. Invasions* 16 2323–2337. 10.1007/s10530-014-0667-4

[B31] Si C. C. Liu X. Y. Wang C. Y. Wang L. Dai Z. C. Qi S. S. (2013). Different degrees of plant invasion significantly affect the richness of the soil fungal community. *PLoS ONE* 8:e85490 10.1371/journal.pone.0085490 PMC3877371 24392015

[B32] Song L. Y. Li C. H. Peng S. L. (2010). Elevated CO2 increases energy-use efficiency of invasive *Wedelia trilobata* over its indigenous congener. *Biol. Invasions* 12 1221–1230. 10.1007/s10530-009-9541-1

[B33] Sun Z. K. He W. M. (2010). Evidence for enhanced mutualism hypothesis: *Solidago canadensis* plants from regular soils perform better. *PLoS ONE* 5:e15418 10.1371/journal.pone.0015418 PMC2972720 21082028

[B34] Taghavi S. Garafola C. Monchy S. Newman L. Hoffman A. Weyens N. (2009). Genome survey and characterization of endophytic bacteria exhibiting a beneficial effect on growth and development of poplar trees. *Appl. Environ. Microbiol.* 75 748–757. 10.1128/AEM.02239-08 19060168 PMC2632133

[B35] Tchinda R. A. M. Boudjeko T. Simao-Beaunoir A.-M. Lerat S. Tsala E. Monga E. (2016). Morphological, physiological, and taxonomic characterization of actinobacterial isolates living as endophytes of cacao pods and cacao seeds. *Microbes Environ.* 31 56–62. 10.1264/jsme2.ME15146 26947442 PMC4791117

[B36] van Kleunen M. Dawson W. Essl F. Pergl J. Winter M. Weber E. (2015). Global exchange and accumulation of non-native plants. *Nature* 525 100–103. 10.1038/nature14910 26287466

