# Supplementary material for: Different Growth Promoting Effects of Endophytic Bacteria on Invasive and Native Clonal Plants
Source: Front Plant Sci. 2016 May 24;7:706. doi: 10.3389/fpls.2016.00706 (PMC4878316; doi:10.3389/fpls.2016.00706)
Supplement: Supplementary file 1 [file Table_1.DOCX]

| **Table S1 Sequence of 16S rDNA from WtEB-JS040 strain** | |
| --- | --- |
| **Position** | **Sequence** |
| 1 | CAGGCCAGGCGCGGGCTGCCTATTAATGCAAGTCGAGCGGACAGATGGGA |
| 51 | GCTTGCTCCCTGATGTTAGCGGCGGACGGGTGAGTAACACGTGGGTAACC |
| 101 | TGCCTGTAAGACTGGGATAACTCCGGGAAACCGGGGCTAATACCGGATGG |
| 151 | TTGTTTGAACCGCATGGTTCAGACATAAAAGGTGGCTTCGGCTACCACTT |
| 201 | ACAGATGGACCCGCGGCGCATTAGCTAGTTGGTGAGGTAACGGCTCACCA |
| 251 | AGGCGACGATGCGTAGCCGACCTGAGAGGGTGATCGGCCACACTGGGACT |
| 301 | GAGACACGGCCCAGACTCCTACGGGAGGCAGCAGTAGGGAATCTTCCGCA |
| 351 | ATGGACGAAAGTCTGACGGAGCAACGCCGCGTGAGTGATGAAGGTTTTCG |
| 401 | GATCGTAAAGCTCTGTTGTTAGGGAAGAACAAGTGCCGTTCAAATAGGGC |
| 451 | GGCACCTTGACGGTACCTAACCAGAAAGCCACGGCTAACTACGTGCCAGC |
| 501 | AGCCGCGGTAATACGTAGGTGGCAAGCGTTGTCCGGAATTATTGGGCGTA |
| 551 | AAGGGCTCGCAGGCGGTTTCTTAAGTCTGATGTGAAAGCCCCCGGCTCAA |
| 601 | CCGGGGAGGGTCATTGGAAACTGGGGAACTTGAGTGCAGAAGAGGAGAGT |
| 651 | GGAATTCCACGTGTAGCGGTGAAATGCGTAGAGATGTGGAGGAACACCAG |
| 701 | TGGCGAAGGCGACTCTCTGGTCTGTAACTGACGCTGAGGAGCGAAAGCGT |
| 751 | GGGGAGCGAACAGGATTAGATACCCTGGTAGTCCACGCCGTAAACGATGA |
| 801 | GTGCTAAGTGTTATGGGGTTTCCGCCCCTTAGTGCTGCAGCTAACGCATT |
| 851 | AAGCACTCCGCCTGGAGAGTACGGTCGCAAGACTGAAACTCAAAGGAATT |
| 901 | GACGGGGGCCCGCACAAGCGGTGGAGCATGTGGTTTAATTCGAAGCAACG |
| 951 | CGAAGAACCTTACCAGGTCTTGACATCCTCTGACAATCCTAGAGATAGGA |
| 1001 | CGTCTCCTTCGGGGGCAGAGTGACAGGTGGTGCATGGTTGTCGTCAGCTC |
| 1051 | GTGTCGTGAGATGTTGGGATAAGTTCCCGCACGAGCGCAACCTTGATCTT |
| 1101 | AAGTTGCCAGCATTCAGTTGGGCACTCCTAAGGTGACTTGCCGTTGGACA |
| 1151 | AC |
