# Supplementary material for: Different Growth Promoting Effects of Endophytic Bacteria on Invasive and Native Clonal Plants
Source: Front Plant Sci. 2016 May 24;7:706. doi: 10.3389/fpls.2016.00706 (PMC4878316; doi:10.3389/fpls.2016.00706)
Supplement: Supplementary file 2 [file Data_Sheet_1.DOC]

Supplementary Material

# Different promoting growth effects of endophytic bacteria on invasive and native clonal plants

Dai Zhi-Cong, FU Wei, WAN Ling-Yun, Cai Hong-Hong,

WANG Ning, Qi Shan-Shan, Du Dao-Lin

*** Correspondence:** Dr. Du Dao-Lin (ddl@ujs.edu.cn) and Dr. Qi Shan-Shan (qishanshan1986120@163.com)

# Supplementary Data

**Identification of aseptic seedlings.**

Tissues of aseptic seedlings were put into 1.5 ml sterilized microcentrifuge tubes, and 800 μl sterilized PBS buffer were added, then were homogenized in a bullet blender. One hundred microliter grinding fluid of each sample was cultured on Luria-Bertani ager media and plates were incubated at 30℃ for 6 days. Also, the total DNA of grinding fluid was extracted to test if there were bacteria using 16Sr-DNA PCR analysis.

**There were no colony in the cultured plates** which were incubated at 30℃ for 6 days (Fig. 1). The 16S-rDNA PCR analysis showed that **there were no bacteria in the aseptic seedlings** (Fig. 2).


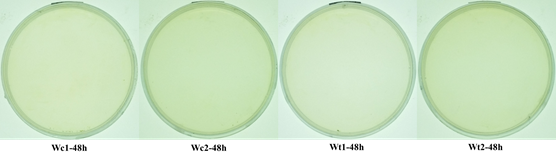


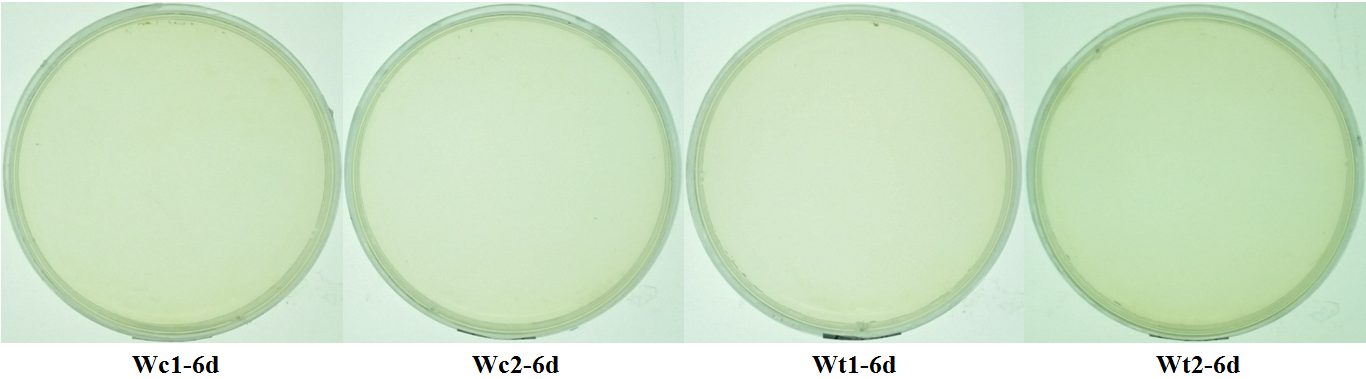


**Fig.1**  Incubated plates of 48 hours and 6 days

(Wc - *Wedelia chinensis*, Wt - *Wedelia trilobata*, two seedlings for each species)


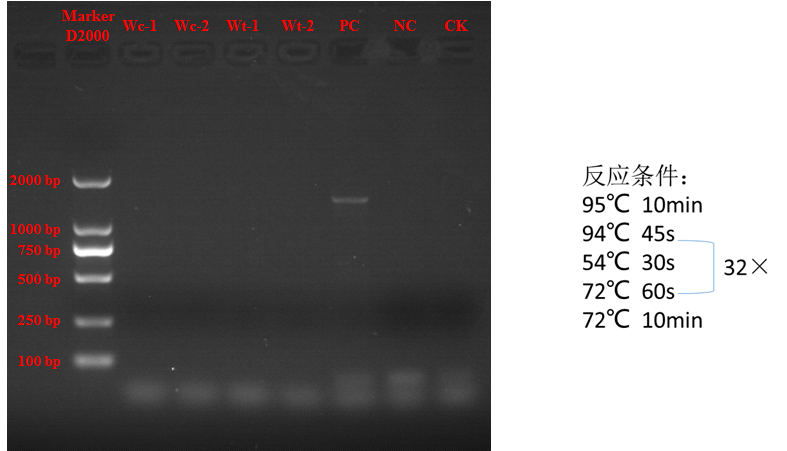


**Fig.2** 16S-rDNA PCR analysis

(Wc - *Wedelia chinensis*, Wt - *Wedelia trilobata*, PC - PCR positive control of JS040, NC - PCR negative control, CK – PBS buffer)

**
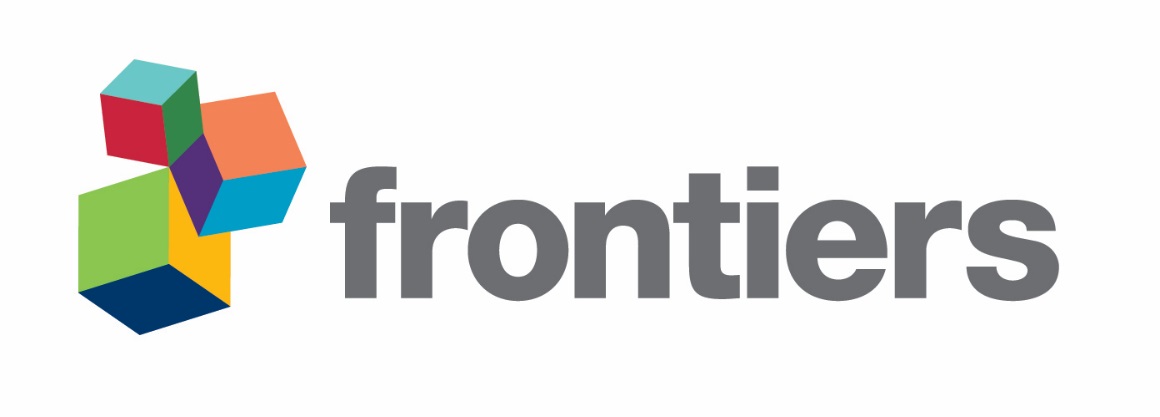
**

**Supplementary Figure 1.** The figure legends are required to have the same font as the main text, 12 point normal Times New Roman, single spaced. Please use a single paragraph for each legend and prepare the figures keeping in mind the PDF layout.
