# Supplementary material for: Different Growth Promoting Effects of Endophytic Bacteria on Invasive and Native Clonal Plants
Source: Front Plant Sci. 2016 May 24;7:706. doi: 10.3389/fpls.2016.00706 (PMC4878316; doi:10.3389/fpls.2016.00706)
Supplement: Supplementary file 3 [file Data_Sheet_2.DOC]

Supplementary Material

# Different promoting growth effects of endophytic bacteria on invasive and native clonal plants

Dai Zhi-Cong, FU Wei, WAN Ling-Yun, Cai Hong-Hong,

WANG Ning, Qi Shan-Shan, Du Dao-Lin

*** Correspondence:** Dr. Du Dao-Lin (ddl@ujs.edu.cn) and Dr. Qi Shan-Shan (qishanshan1986120@163.com)

# Supplementary Data

**Identification of** **colonization of JS040 in aseptic seedlings.**

After 21 days of inoculation with JS040 (*E*^+^) or without JS040 (*E*^-^) (for the detailed inoculation method, please see the "Aseptic Seedlings and Aseptic Culture System" and "Endophyte Experiments" in the Method section of manuscript), tissues of seedlings were put into 1.5 ml sterilized microcentrifuge tubes, and 800 μl sterilized PBS buffer were added, then were homogenized in a bullet blender. One hundred micro liter grinding fluid of each sample was coated on Luria-Bertani ager media and plates were incubated at 30℃ for 6 days, 100 μl sterilized PBS buffer was also coated as control treatment (CK).

**There were no colony in the cultured plates of CK and *E*^-^****treatments, however, there were bacteria clones in the *E*^+^ treatments** (Fig. 1).


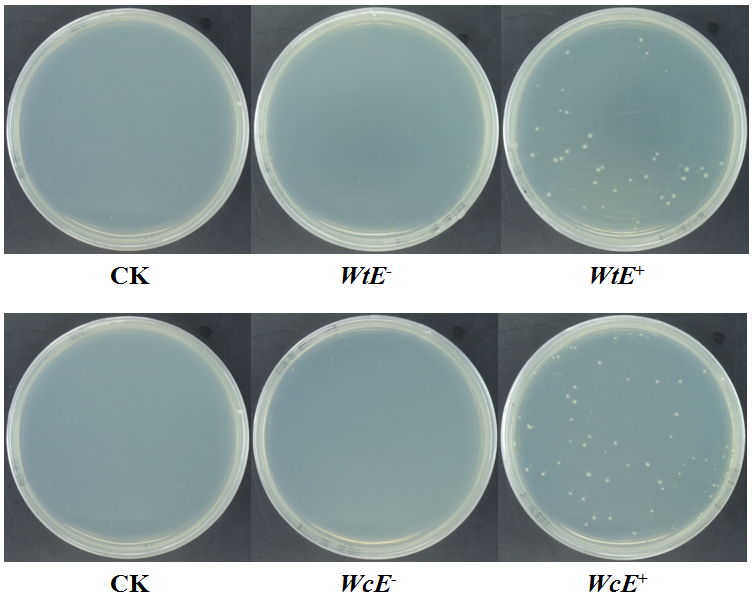


**Fig.** **1** Incubated plates of aseptic seedlings after inoculation of WtEB-JS040 for 21 days

(CK- PBS buffer, Wc - *Wedelia chinensis*, Wt - *Wedelia trilobata*,

E^-^/E^+^- seedlings inoculated without/ with WtEB-JS040)

Then, three clones from the media plates of each plant species were randomly selected and amplified using 16S-rDNA primers Bac8F (5'-AGAGTTTGATCCTGGCTCAG-3') and 1492R (5'-GGTTACCTTGTTACGACTT-3'), then sent to Sangon Biotech (Shanghai) Co., Ltd. (China) for DNA sequencing. The 16S sequences of clones from seedlings sample of each plant species were aligned and clustered with the sequences of JS040 strain using Kimura method in DNAMAN software.

**The 16S sequences alignment revealed desirable identity (98.67%, Fig. 2), showing these clones from seedlings sample of each plant species originate from JS040. Therefore, these results showed that JS040 had** **colonized in the aseptic seedlings.**

**Fig. 2** The alignment of 16S sequences of clones from seedlings sample of each plant species with JS040 strain (Wc / Wt - clones of JS040 colonization in *Wedelia chinensis* or *Wedelia trilobata*)

**
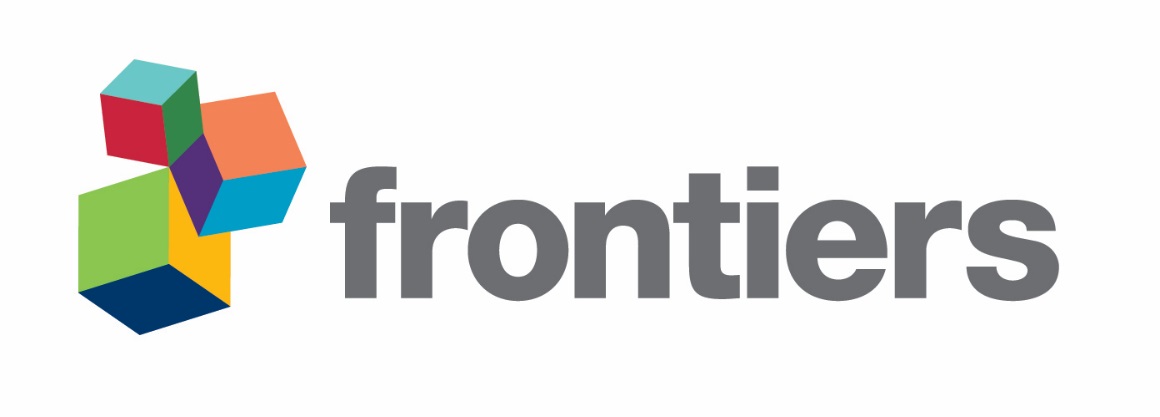
**

**Supplementary Figure 1.** The figure legends are required to have the same font as the main text, 12 point normal Times New Roman, single spaced. Please use a single paragraph for each legend and prepare the figures keeping in mind the PDF layout.
