# Supplementary figures and images for: Different Growth Promoting Effects of Endophytic Bacteria on Invasive and Native Clonal Plants
Source: Front Plant Sci. 2016 May 24;7:706. doi: 10.3389/fpls.2016.00706 (PMC4878316; doi:10.3389/fpls.2016.00706)

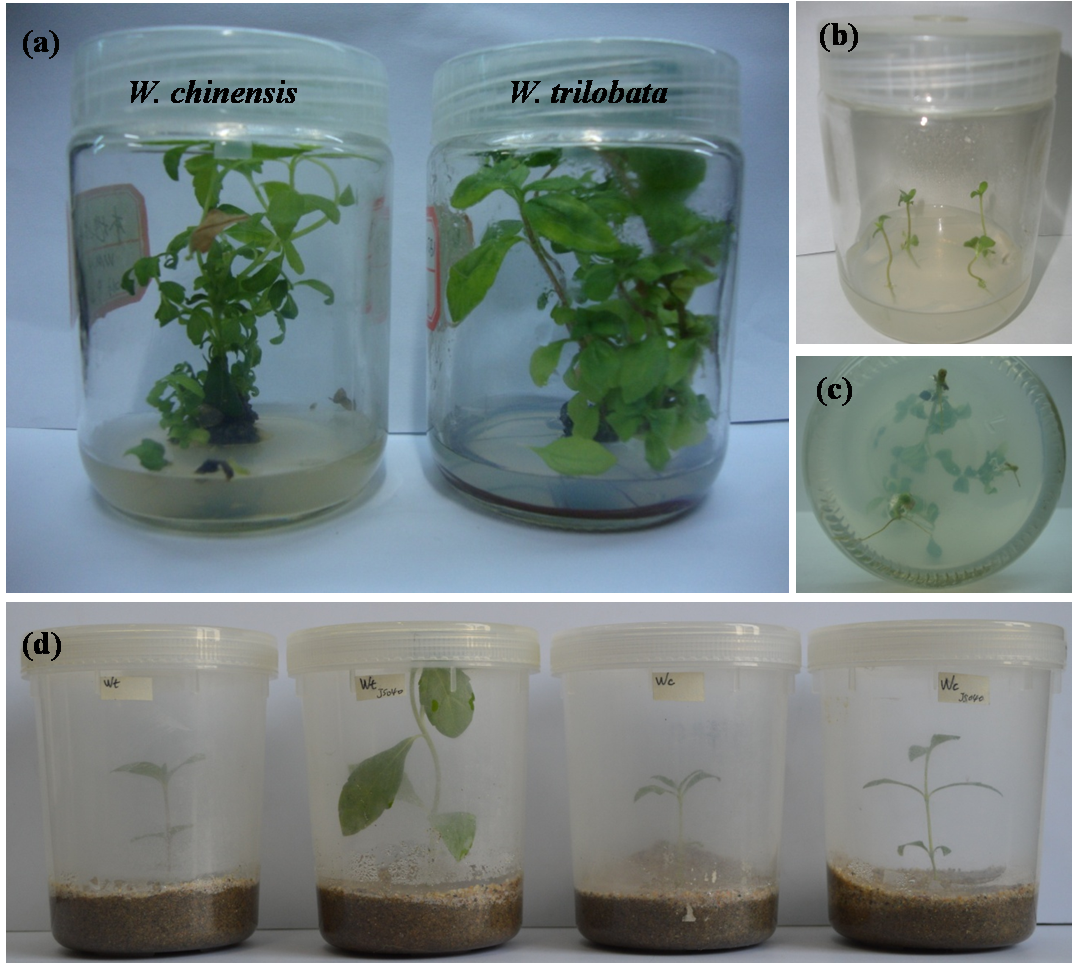

Supplement: FIGURE S1 — Aseptic culture system for endophytes research. (A) multiple axillary buds, (B) apical shoots cut from axillary buds, (C) aseptic seedling with roots, (D) aseptic seedling system. [file Image_1.TIF]

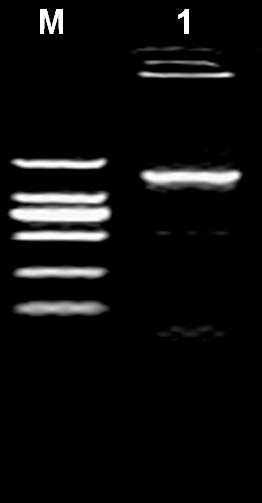

Supplement: FIGURE S2 — Amplification of 16S-rDNA from WtEB-JS040 strain. Lane M, DL2000 LADDER; Lane 1, 16S-rDNA fragment amplified by PCR. [file Image_2.TIF]
